# An Integrative, Systematic Review Exploring the Research, Effectiveness, Adoption, Implementation, and Maintenance of Interventions to Reduce Sedentary Behaviour in Office Workers

**DOI:** 10.3390/ijerph15122876

**Published:** 2018-12-15

**Authors:** Bradley MacDonald, Xanne Janssen, Alison Kirk, Mhairi Patience, Ann-Marie Gibson

**Affiliations:** School of Psychological Sciences and Health, University of Strathclyde, 16 Richmond Street, Glasgow G1 1XQ, UK; xanne.janssen@strath.ac.uk (X.J.); alison.kirk@strath.ac.uk (A.K.); MhairiPatience1@hotmail.com (M.P.); annmarie.gibson@strath.ac.uk (A.-M.G.)

**Keywords:** sitting time, sedentary, occupational, office workers, RE-AIM, translation, evaluation, review

## Abstract

Sedentary behaviour is associated with poor health outcomes, and office-based workers are at significant health risk, as they accumulate large proportions of their overall sitting time at work. The aim of this integrated systematic review was to collate and synthesize published research on sedentary behaviour interventions in the workplace that have reported on at least one an aspect of the reach, effectiveness, adoption, implementation, and maintenance (RE-AIM) framework. Studies were included if they involved adult office workers, were conducted in an office setting, and changes in sedentary behaviour had been measured as a primary outcome. Five electronic databases were searched yielding 7234 articles, with 75 articles (61 individual interventions) meeting the inclusion criteria. Reach indicators were the most frequently reported RE-AIM dimensions, which were reported on average 59% of the time. Efficacy/effectiveness was the second most reported dimension at 49% reporting across all of the indicators. Implementation indicators were reported an average of 44% of the time, with indicators of adoption and maintenance reported as the lowest of all indicators at 13% and 8%, respectively. Recommendations are provided to improve reporting across all RE-AIM dimensions, which is an important first step to enable the effective translation of interventions into real world settings.

## 1. Introduction

Sedentary behaviour (SB), or sitting time, is associated with an increased risk of chronic diseases, such as metabolic syndrome, cardiovascular disease, and diabetes mellitus, in addition to increased all-cause mortality in adults [1,2,3]. Despite the health risk, representative samples indicate that the prevalence of sedentary behaviour is high in Western adults (between 6.8 and 11.2 h/day) [4,5,6]. Research suggests that office-based workers are at significant health risk, as they accumulate large proportions of their overall sitting time at work [7,8,9]. The global prevalence of occupational sitting will likely continue to rise as the labour market continues to shift towards computerised employment [10]. Consequently, the United Kingdom has developed guidance for employers in order to promote the avoidance of prolonged periods of sedentary work [11].

There has been an increase in interventions targeting sedentary office workers [12,13,14,15], and a number of reviews of the intervention work have followed [16,17,18,19]. The majority of these reviews have provided an evaluation of these interventions in relation to indicators of “efficacy” [16,17,18,19]. However, there has been growing critique suggesting that, although indicators of efficacy are important to assess, there is little understanding of the additional indicators, which may help to understand the potential for successful translation and future real-world implementation [20]. Critics argue that other indicators that facilitate an understanding of generalisability and translation are equally important to evaluate, particularly if these additional indicators impact the success of future implementation, and consequently the potential public health impact of a given intervention [20,21].

The RE-AIM evaluation framework is one of several existing methods used to evaluate or report on the additional indicators that could influence the future external validity of an intervention. Glasgow et al. (1999) [22] proposed five dimensions in which these indicators sit—*reach*, *efficacy/effectiveness*, *adoption*, *implementation*, *and maintenance*. Reach is defined as the absolute number, proportion, and representativeness of individuals who are willing to participate in a given initiative. Efficacy/effectiveness refers to the impact of an intervention on the relevant outcomes, including potential adverse effects, quality of life, and economic outcomes. Adoption, within RE-AIM, is the absolute number, proportion, and representativeness of the settings and intervention agents who are willing to initiate a program. Implementation refers to the intervention agents’ (e.g., research teams) fidelity to the various elements of an intervention’s protocol. This includes consistency of delivery as intended, and the time and cost of the intervention. The maintenance dimension is concerned with both the setting and individual level. At the setting level, maintenance is the extent to which a program or policy becomes institutionalised or part of organisational practices and policies. At the individual level, maintenance has been defined as the long-term effects of a program on outcomes from six months onwards from the most recent contact [22,23].

Glasgow et al. (2004) [23] further explains that evaluating interventions over the five dimensions of the RE-AIM framework will help to facilitate an understanding of the potential external validity and public health impact of an intervention. This type of reporting is critically important as we move on a continuum from understanding an intervention effect produced under controlled conditions, towards implementation under real world conditions [21]. To date, no systematic reviews on sedentary behaviour interventions in office workers have been conducted using the RE-AIM framework. Therefore, the aim of the current study is to conduct a systematic review of sedentary behaviour interventions in the workplace focusing on the RE-AIM dimensions (reach, effectiveness, adoption, implementation, and maintenance). The review aims to gain an understanding of the proportion of RE-AIM indicators that are reported in the literature so as to identify whether gaps in reporting exist, which indicators are underreported, and which existing methods may be useful in collecting data on underreported indicators.

## 2. Methods

In order to capture published literature reporting on any dimension of the RE-AIM framework, an integrative, systematic review approach was used. The integrative methodology is specifically designed to facilitate the inclusion of a broad range of research designs, both qualitative and quantitative, so as to comprehensively understand a given phenomenon [24].

### 2.1. Search Strategy

Studies were included if they involved adult office workers, were conducted in an office setting, and if changes in sedentary behaviour had been measured (objectively or subjectively) as a primary outcome of the study. No limitations were placed on the design of the study. The inclusion/exclusion criteria and search terms were developed through scoping searches. The review team used PICOS criteria (population, intervention, comparators, outcome, and setting) to facilitate this process (Table 1). The search terms were used to search five electronic databases (MEDLINE (Ovid platform), PsycINFO, SPORTDiscus, Business Source Complete, and OPEN Grey), and searching was completed on 7 December 2017.

### 2.2. Screening Process

**Figure 1:**
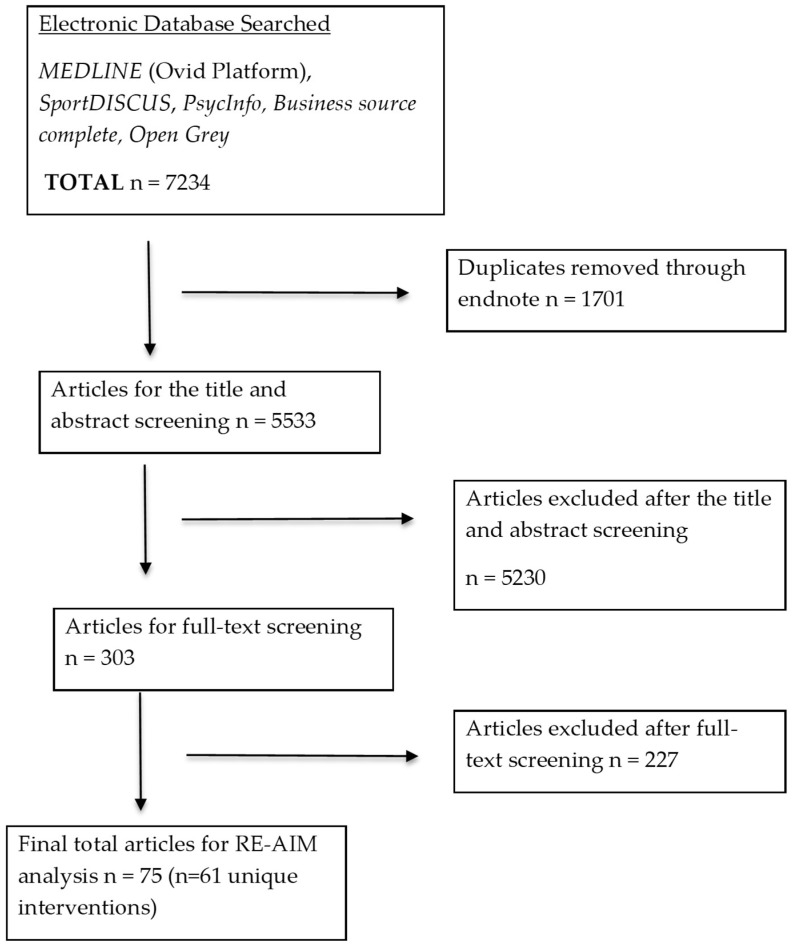
Flow diagram of studies included in the review. RE-AIM—reach, effectiveness, adoption, implementation, and maintenance.

The retrieved articles (*n* = 7234) were exported into EndNote (Clarivate Analytics, Philadelphia, PA, USA) so as to remove duplicates. After the removal of the duplicates, a total of 5533 articles were left. These articles were then exported into Covidence (Covidence, Melbourne, Australia) for screening. Covidence is an online platform that is designed to enhance the reliability of systematic reviews by facilitating organisational that which enhance the rigour within the screening process. The platform also facilitates the blinding of the screening process between reviewers. Double screening of the studies was carried out at two stages, namely: title/abstract and full text. At the end of each stage, two reviewers (B.M. and M.P.) met to discuss the disagreements. Cohen’s Kappa calculations were done for the title and abstract (0.96), and for the full text (0.97). The studies that could not be agreed upon were brought to a third member of the review team (X.J.) and were discussed. On all occasions, a final decision was agreed upon by all parties. Figure 1 highlights this process.

### 2.3. Data Extraction

The data was extracted using a combination of two validated RE-AIM coding sheets [23,25,26,27]. The combination of the two sheets facilitated in the coding of information across all five dimensions of the RE-AIM framework, looking at 28 individual indicators from each intervention. The alignment of these indicators to each dimension of the RE-AIM framework is noted below.

#### 2.3.1. Reach

The items from the extraction tool that facilitated in reporting on the potential reach of an intervention included the following: the method used to identify the target population, inclusion criteria and exclusion criteria, use of qualitative methods to understand reach or recruitment, sample size, participation rate, and sample representatives. The participation rate was calculated based on the reported number of participants, divided by the number of eligible participants exposed to recruitment. The sample representativeness information was extracted if an intervention reported the demographics of both the participants and eligible non-participants.

#### 2.3.2. Efficacy/Effectiveness

The efficacy and effectiveness items included the following: assessment of the effect on outcomes at shortest assessment point, imputation procedures reported, the presence of quality of life measure, effects at longest follow-up, use of qualitative methods to understand outcomes, and percent attrition or dropout rate. If the attrition rate was not directly reported, it was calculated based on the participant numbers at randomization, as compared to the participant numbers at shortest assessment point.

#### 2.3.3. Adoption

The items that were extracted for *adoption* related to both the setting and participants. Specifically, the extent to which a study reported; *the method of identifying target agent*—an agent should be identified regardless of the type of intervention (e.g., device-based or consultation approach); *level of expertise of delivery agents* (e.g., was specific training or level of understanding or influence reported for different intervention agents)—may be less relevant in device based interventions; *inclusion and exclusion criteria for target agent*—relevant for all intervention types; *the adoption rate (e.g., number of companies who took part/number of companies who were approached)*—relevant for all intervention types; *comparison of settings/participants of adoption vs. non-adoption settings* (e.g., demographic or environmental differences between adoption of program/intervention vs. non-adoption)—relevant for all intervention types; *and use of qualitative methods to understand either adoption at setting level and staff participation*—relevant for all intervention types.

#### 2.3.4. Implementation

Information relating to the implementation that was extracted and reported on. Specifically, *the intervention type* (e.g., individual component vs. multi-component) *and intensity*. With no specific guidance on a measure of intensity, the review team judged the reporting of intensity based on the reporting of the length of the intervention, as well as components of the intervention. Further items included the following: the extent the protocol was delivered as intended (e.g., did the intervention achieve its intended implementation goal or did protocol need to be adapted); a measure of cost (e.g., monetary or time commitment); and use of qualitative methods to understand the implementation of the study.

#### 2.3.5. Maintenance

Maintenance was assessed using the following three items: was an individual’s behaviour assessed at least six months following the completion of the intervention; is the program still in place, was the program modified, and use of qualitative methods to understand long-term effects.

All of the relevant information was extracted and coded in an excel spreadsheet by two reviewers (B.M. and M.P.), with each researcher extracting half of the papers. Upon the completion of the extraction, each of the 28 items were colour coded green if the information was presented, or red if the information was not presented. All of the data extraction was then double checked by a third member of the review team (X.J.) so as to enhance reliability.

### 2.4. Quality Assessment

Because of the broad range of study designs and the use of the RE-AIM reporting item for data extraction and reporting, no further assessment of the study quality was performed.

## 3. Results

### 3.1. Study Selection

The initial searches identified 7234 articles, and after title and abstract screening, 303 full text articles were screened. Of these, 75 articles representing 61 individual interventions were included in the review (Figure 1).

### 3.2. Characteristics of Identified Articles

Table 2 describes the characteristics of the identified articles. It is important to understand the distinction between the articles and interventions from this point forward in the review. The results of 10 interventions were reported in more than one article. This information has been brought together in order to understand the reporting of all of the indicators across the dimensions of the RE-AIM framework. This method has been used in other RE-AIM reviews for the same purpose [26,28]. In total, there were 75 included articles in the review, representing 61 individual interventions. Table 2 identifies which articles are from the same intervention. Of the 61 interventions, 23 interventions were completed in North America, 22 in Europe, 15 in Australia, and 1 in South America. The integrated review approach facilitated a large variety in both the study design and outcome measurement method. Of the 75 published articles, 39 reported controlled designs (both randomised and non-randomised), which was the most frequent. A total of fifteen articles reported pre- and post-test experimental designs; seven reported qualitative designs, six of which were reported as natural experiments; five reported quasi experimental designs, one of which was a cross sectional design; one reported mixed methods design; and one reported descriptive design. The duration of the interventions that were included ranged from one day to 12 months, with 20 interventions reporting less than 7 weeks, 25 interventions reporting 2–4 months, nine interventions reporting 4–9 months, and five interventions reporting 12 months. Two interventions did not report an intervention duration. In total, 17 individual data collection methods were used to measure sedentary behaviour (SB). Objective measures of SB were used in 39 interventions, with the most common being ActivPAL (*n* = 20). Other objective measures included accelerometery, video analysis, and objective proxy measures. Subjective measures of SB were used in 31 interventions, with the most common type being a questionnaire (*n* = 23). Other subjective methods included interview, focus group, diary/log, and open-ended questions. It should be noted that the number of SB outcome measures does not exactly equal the number of included interventions, as a result of nine of the 61 interventions using both objective and subjective measures of SB.

### 3.3. Percentage Reporting across RE-AIM Dimensions

**Figure 2:**
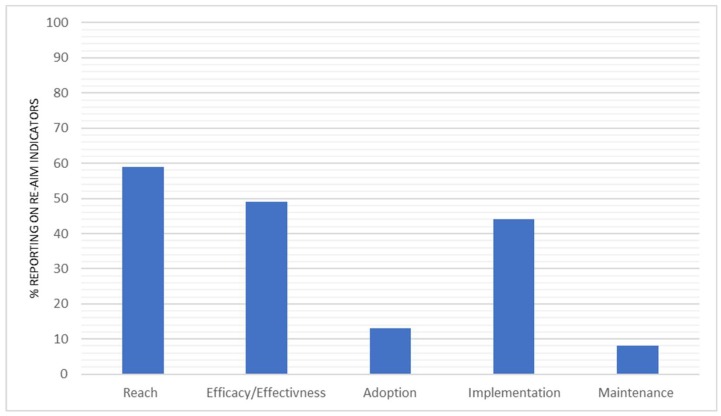
The total proportion of reporting across all indicators within each RE-AIM dimension.

The total percentage of reporting across all of the indicators within the individual RE-AIM dimension is represented in Figure 2. Reach indicators were reported on average 59% of the time. Efficacy/effectiveness was reported at 49% across all of the indicators. Implementation indicators were reported an average of 44% of the time. The overall percentage of interventions reporting on the indicators of adoption and maintenance indicators were 13% and 8%, respectively. A full break down of reporting across all of the indicators for individual studies is available in Supplementary Tables S1 and S2.

### 3.4. Reach

**Figure 3:**
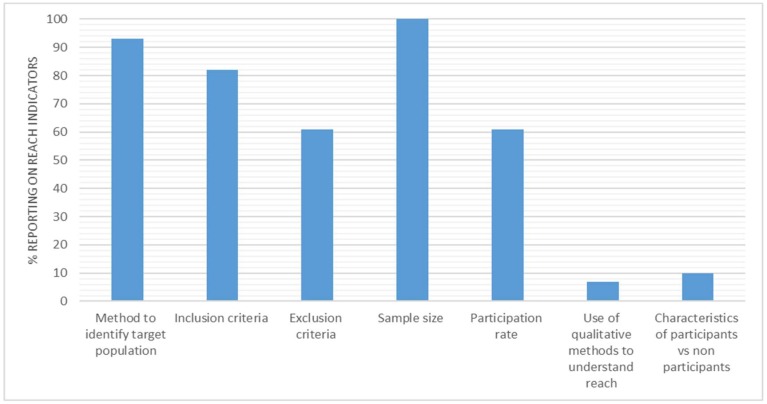
Percentage of studies reporting reach indicators.

There was a significant variation between the reach indicators (Figure 3), with a high reporting of three indicators, namely, identifying target population (*n* = 57, 93%), inclusion criteria (*n* = 50, 82%), and sample size (*n* = 61, 100%). The reporting of exclusion criteria and participation rate were lower, with both being reported at 61% (*n* = 37). There was low reporting for the characteristics of participants vs. non-participants (*n* = 6, 10%), and for the use of qualitative methods to understand reach (*n* = 4, 7%).

### 3.5. Efficacy/Effectiveness

**Figure 4:**
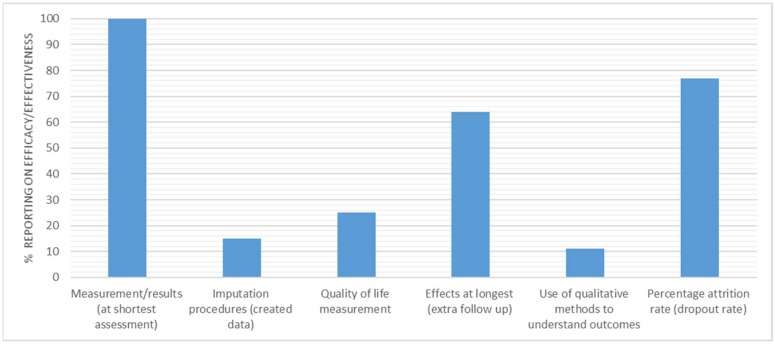
Percentage of interventions reporting efficacy/effectiveness indicators.

Figure 4 illustrates the percentage of reporting for individual efficacy/effectiveness indicators. High reporting was noted across several indicators, including the following: the measure of primary outcome at the shortest assessment point (*n* = 61, 100%), and the percent attrition rate (*n* = 47, 77%). The measurement of the primary outcome at extra follow up points was reported for 39 interventions (64%). The reporting dropped significantly for the remaining three indicators, with 15 interventions (25%) reporting on quality of life measurement, nine interventions (15%) reporting imputation or intention to treat analysis, and seven interventions (11%) reporting use of qualitative methods to understand outcomes.

### 3.6. Adoption

**Figure 5:**
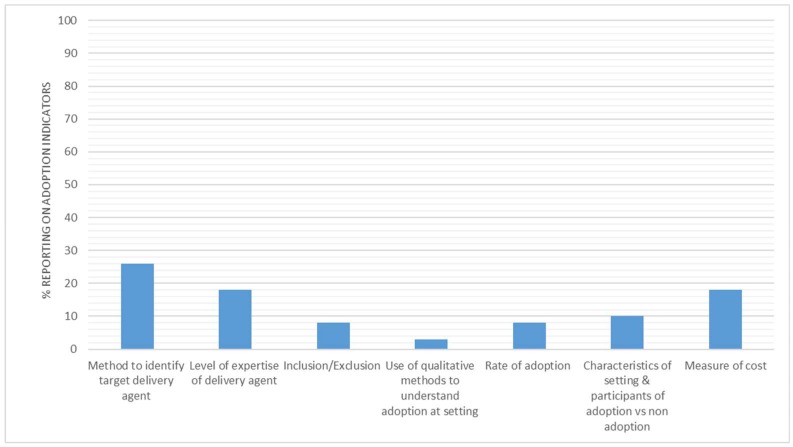
Percentage of interventions reporting adoption indicators.

Figure 5 illustrates the percentage reporting for individual adoption indicators. In total, 16 interventions (26%) reported methods to identify delivery target agent, 11 interventions (18%) reported the level of expertise of the delivery agents, and five interventions (8%) provided inclusion/exclusion criteria concerning adoption at the setting level. Furthermore, five interventions (8%) reported a rate of adoption at the setting level, two interventions (3%) reported the use of qualitative methods to understand adoption, and six interventions (10%) reported differences in characteristics (either participant or setting) of adoption vs. non-adoption.

### 3.7. Implementation

**Figure 6:**
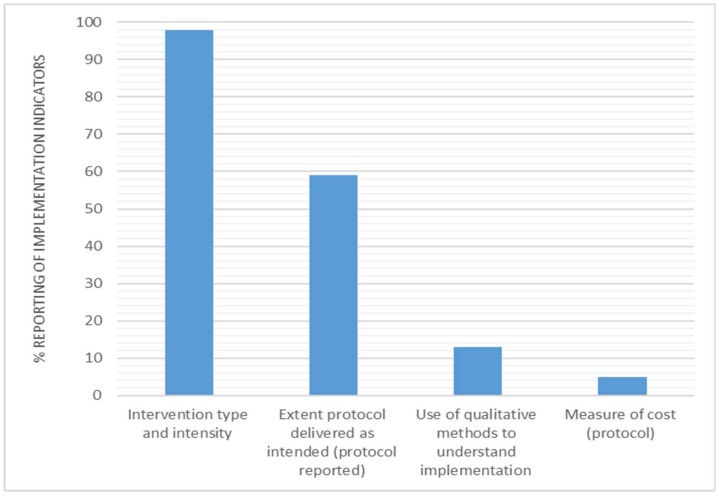
Percentage of interventions reporting implementation indicators.

Figure 6 illustrates the reporting for implementation. The most commonly reported indicator was the intervention type and intensity (*n* = 60, 98%). In total, 36 (59%) interventions reported on the extent the protocol was delivered as intended, and eight interventions (13%) used qualitative methods to understand implementation. Finally, a measure of cost (protocol) was reported in three interventions (5%).

### 3.8. Maintenance

**Figure 7:**
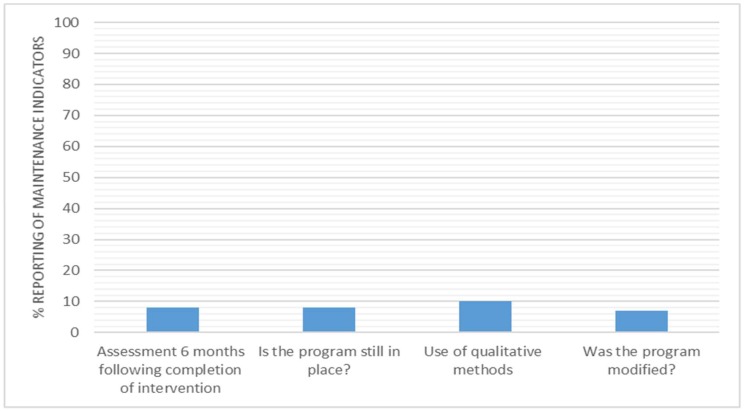
Percentage of interventions reporting maintenance indicators.

Concerning individual indicators of maintenance (Figure 7), five interventions (8%) reported on an individual behaviour assessment at least six months following the completion of the intervention; five interventions (8%) reported whether the program is still in place, six interventions (10%) reported the use of qualitative methods to understand setting level institutionalization, and four interventions (7%) reported if the program was modified.

## 4. Discussion

The purpose of this review is to provide an understanding of the depth of reporting of indicators across the RE-AIM dimensions. Previous systematic reviews have investigated the effectiveness of workplace SB interventions [16,17,18]. However, to the authors’ knowledge, this is the first systematic review focusing on RE-AIM reporting in office-based SB interventions. This review is the first to synthesise a breadth of the evidence in the field, with a focus on the reporting of indicators important to the future implementation and translation of interventions.

The reach indicators were the most frequently reported of all of the RE-AIM dimensions; reported on average 59% of the time. Efficacy/effectiveness was the second most reported dimension at 49% reporting across all of the indicators. The implementation indicators were reported an average of 44% of the time. The overall percentage of studies reporting on the indicators of adoption and maintenance were the lowest of all of the RE-AIM framework indicators at 13% and 8%, respectively. The results revealed that 10 of the 28 indicators were reported more than 50% of the time however, and the remaining 18 indicators were reported less than 30% of the time, revealing a distinct contrast in the indicators that are routinely reported in interventions. In light of this result, the research team has focused the discussion primarily on the indicators or indeed the whole dimensions that have been “under-reported” or have been reported for less than 30% of the interventions. The discussion firstly presents specific methods used to capture the data from underreported indicators of RE-AIM; and secondly, provides future considerations and recommendations for collecting the data of under reported RE-AIM indicators. This is done in order to facilitate improved reporting (success and failure) across the RE-AIM dimensions, so as to improve our evaluation of generalisability and potential translation of interventions, as well as the potential for the public health impact of interventions [20,21,101].

### 4.1. Reach

The distinct contrast in reporting is evident in reach (Figure 2). Some indicators of reach are well reported across the included interventions, such as, a method to identify the target population (*n* = 57, 93%) or inclusion criteria (*n* = 50, 82%). However, reach indicators such as representativeness of participants vs. non-participants (*n* = 6, 10%), and use of qualitative methods (*n* = 4, 7%) are underreported. Nevertheless, interventions such as those of De Cocker et al. (2016, 2017) [55,56,57] and Bort-Roig et al. (2014) [33] highlight the methods for reporting on these indicators specifically.

De Cocker et al. (2016) delivered computer-tailored advice to influence sitting behaviour [56,57]. To report on the representativeness of participants vs. non-participants, the authors utilised the already available health information of the office employees that did not participate, and did a comparative analysis to the demographics of the workers who participated [55,56,57]. In De Cocker’s intervention, the office workers who were less educated were less likely to participate, therefore, an educational element may be critical in order to engage less educated office workers [56,57]. This example highlights how information on representativeness can provide further insight into how to best target intervention strategies.

Additionally, the data collected by Bort-Roig et al. (2014) used a qualitative methodology to facilitate an understanding of the participant uptake [33]. In the study, they interviewed the implementation team regarding their perceptions of factors that impacted on uptake within the study. They then triangulated the interview results with the participant surveys that rated the extent to which the uptake strategies were used [33]. This triangulation process facilitated understanding of reach, giving context to the factors that influenced the study population.

These two studies highlight methods that can be used to improve on the reporting of indicators of reach. Each method improved the understanding of the factors, which may impact on the future implementation and translation of the studies, and therefore, have a potential public health impact.

### 4.2. Efficacy/Effectiveness

As with reach, there are distinct differences in the indicators of efficacy/effectiveness that are routinely reported (Figure 3). The reporting of measure/results (at shortest assessment) (*n* = 61, 100%), effects at longest (extra follow up) (*n* = 39, 64%), and the percent attrition rate (dropout rate) (*n* = 47, 77%) were significantly higher than the quality of life measurement (*n* = 15, 25%) and use of qualitative methods or data to understand outcomes (*n* = 7, 11%), both of which were underreported.

SB is associated with the additional health related outcomes that may affect the “quality of life” of the participants, including, back, shoulder, and neck pain [102,103,104], and a variety of psychological issues, for example, depression [105], distress [106], and anxiety [107]. Therefore, these outcomes are also important to measure so as to improve our understanding of the association, and to monitor negative unintended outcomes. Importantly, the measurement of additional quality of life outcomes has the potential to strengthen the arguments for the importance of reducing office-based SB. For example, the methods utilised in the Pronk et al. (2012) [89] intervention “take a stand” provided an example of reporting quality of life measurement [89]. In the intervention, the research team administered validated questionnaires to collect data related to additional work-related outcomes (pre- and post-intervention), which facilitated reporting in relation to the quality of life indicator. The results showed that reductions in the sitting time were significantly associated with reductions in upper back and neck pain, fatigue, confusion, and total mood disturbance [89]. In this example, the measurement of the additional outcomes provided evidence that the intervention was not negatively affecting other related health conditions. This type of measurement may help to increase our understanding of other additional benefits of reducing office-based SB.

Hardgraft et al. (2017) [47] used interviews and focus groups to facilitate in understanding how additional factors impacted on the effectiveness of the strategies used in the study [47]. The authors found that specific at work “job tasks” were barriers to behaviour change, however “social support” was a facilitator [47]. Using qualitative methods improved how Hardgraft et al. (2017) understood how behaviour change occurred, and may be critical for improving efficacy/effectiveness in future iterations of the study [47].

It is clear that reporting on additional indicators of RE-AIM fostered a more holistic understanding of the real impact of the interventions. This information may now be used to help improve the future implementation and translation of the research into different settings.

### 4.3. Adoption

This review has highlighted the underreporting of all of the indicators of the adoption dimension (Figure 4). This is an interesting finding that, on face value, appears to give evidence of poor reporting on setting level indicators. However, a limited number of interventions were implemented across multiple settings (*n* = 16, 26%) in this review. Most of the included interventions were implemented in one setting only and on a relatively small scale (67%, <50 participants); this illustrates a clear gap in the literature.

This review gives further evidence that there is a barrier to translating research from small scale SB interventions to larger scale effectiveness trials [16,101]. The result of this review suggests that one barrier to translation may be the under reporting of indicators that would facilitate effective translation. However, resources, for example time and money, are also significant barriers that often result in pragmatic decision making with respect to the scale of implementation [20,21,101]. The solution to these significant barriers may lie in our engagement with additional stakeholders in workplace health. Companies continue to increase resources in order to improve employee health and wellbeing, as they increasingly understand the relationship between productivity and health status [108,109,110]. However, workplace health promotion programs are often not informed by evidence, and a recent review suggests that programs that are informed by research have more potential to yield positive results [111]. Therefore, a more “practice based” [21] approach, in which researchers work directly with workplace health promotion stakeholders, would bring together both the evidence-based knowledge and resources needed to effectively translate on a larger scale [21]. For this approach to be successful, understanding and addressing the potential barriers to working directly with companies would be important. For example, with new data protection regulations being implemented, one barrier to overcome may be the companies’ willingness to share/collect the health data of employees, with potential concerns that, if misused, it may bring harm to their employees [112,113]. However, if the relationship is nurtured, and concerns are mediated, the approach could help embed public–private partnerships at earlier stages of research. This will help to build stronger practice-based relationships as projects develop [114]. The approach could also circumvent funding bodies, which can be reluctant to fund scaled up trials, which are seen as less “scientifically pure” [115]. Although trade-offs in experimental design may be made, this more pragmatic “practice-based” [21] approach would produce evidence that more accurately reflects the conditions in which it is expected to be applied [20,21,116,117].

Of the 26% of the interventions implemented across multiple settings, there are none that reported all of the adoption indicators. However, there are examples of quality reporting of some individual indicators. For example, Brakenridge et al. (2016, 2017), who had the highest reporting in the review (21 of 28 indicators), reported four of the seven indicators of adoption [36,37]. In Brakenridge et al. (2017), the researchers interviewed members of the implementation team and conducted focus groups with participants in order to understand the differences in implementation across settings [37]. Qualitative findings revealed that there were differences in the role model influence and management engagement across settings, and this may have impacted on variations in the intervention effects across settings [36,37]. Collecting this information may help to improve future translations of this type of intervention. Additionally, when reporting the level of expertise of the delivery agent, Aittasalo et al. (2017) [29] explained the training process of the delivery agents, including the number of hours spent training face to face [29].

These two examples highlight that, when implementation across settings is done in office-based SB interventions, the collection and dissemination of the indicators of adoption enhances our understanding of the translational issues critical to the improvement of future implementation.

### 4.4. Implementation

The reporting of the indicators relating to the implementation dimension was mixed (Figure 5). Nearly all of the studies included in this review (*n* = 60, 98%) reported on the type of intervention and intensity by explaining the intervention activities in detail, and many studies (*n* = 36, 59%) reported on the extent the protocol was delivered as intended (development of a protocol). There was minimal reporting on the indicators which that are important for obtaining similar effects in future iterations of the study. These would include indicators that, for example, question whether the protocol was delivered by the implementation team as the intended? What aspects of the intervention were more or less effective than others? What was the cost (e.g., time commitment or monetary) to implement the intervention? Reporting on these indicators is critical to understanding which specific behaviour change strategies were successfully implemented and caused an effect within a study, and which were less successful. For example, Bort-Roig et al. (2014) [33] found, using both questionnaire and focus group data, that walk–talk meetings and lunch walking groups were rarely utilised within the intervention, and sitting time and step count logging were the most critical enabler of behaviour change. These results would be important to consider for the future implementation of this intervention, and may even trigger adaptations to the less successful strategies, potentially improving the potential public health impact of the study [33].

### 4.5. Maintenance

There was under-reporting of all of the indicators related to the maintenance dimension of RE-AIM (Figure 6), averaging just 8% overall (Figure 1). Two of the indicators assessed whether studies report on (a) if the program is still in place and (b) if the program was modified. These two indicators were only reported 8% (*n* = 5) and 7% (*n* = 4), respectively; however, Parry et al. (2013) [87] exemplified how this type of information could easily be reported, explaining, “The trial was ended due to the lack of further organisations willing to participate within the two-year data collection period” [87]. A third indicator looked for reporting on the follow up measurement six months post intervention. This indicator was also underreported (*n* = 5, 8%). This result is indicative of the fact that 41 of included studies were less than four months in length. From this analysis, it is clear there is a need for longer follow up periods. Interestingly, all of the studies that reported six-month follow-up data did so using self-report methods. Although self-report has its limitations, these results indicate that it may be best placed to pragmatically evaluate the long-term effect, which is vital to understand if long term public health impact is the objective. The six studies that reported on the final indicator of maintenance utilised qualitative methods in order to understand the setting level institutionalisation. For example, in Cifuentes et al. (2015) [44], the reporting highlighted significant barriers to maintaining change in the long term and highlighted areas, which would need to be adapted for the successful future uptake of the intervention [44].

### 4.6. Indicators of Cost

There were two indicators of cost within RE-AIM. Both referred to a measure of cost of implementation either at the individual level (implementation) or at setting level (adoption). Both of the indicators were under-reported, with measure of cost within implementation reported in just two interventions (3%), and measure of cost within adoption reported in 11 interventions (18%). These studies did report elements of cost, however, there was no clear example of a robust method used to fully understand the “cost” of an intervention. There is the potential to measure cost, however gaining transparency may require the development of methodology specific to office-based health promotion, which can articulate the costs incurred balanced with the benefits gained.

### 4.7. Recommendations for Future Reporting

In light of the significant gaps in reporting, the research team have created specific recommendations for the improved future reporting of office-based SB interventions (Table 3). Process evaluation is a critical part of any intervention study, however our review highlights a clear gap in the reporting of indicators that informs this practice [20]. The recommendations highlight that the RE-AIM framework may prove useful in providing a framework for collecting this breadth of process data or information. Additionally, it is clear from the recommendations that this process would require a mixed methods approach [118,119]. Using appropriate methods to capture the necessary data is the first step to both, improved translation, and population level impact.

Reporting on this breadth of indicators would often lead to the publication of a process evaluation, and this would be recommended in order to provide the capacity for reporting over so many indicators. The collection of data on under reported indicators can be done retrospectively [120]. However, it would be seen as best practice to imbed the necessary data collection methods in the initial study design, so as to inform the process evaluation [20]. Both retrospective and embedded process evaluation take careful and considered planning, however the RE-AIM recommendations would prove useful in both cases.

## 5. Strengths and Limitations

A key strength of the review is that it is the first review to look at a large proportion of published interventions that have been done targeting office based sedentary behaviour, in order to understand the state of reporting for effective future translation. This may be crucial to understand, as future population level impact relies on successful translation. Additionally, using the RE-AIM framework enabled an in-depth and critical analysis of the individual papers. This critical approach has facilitated the creation of specific and considered recommendations to enhance future intervention reporting within office-based sedentary interventions. Furthermore, the use of software tailored for reviews enabled quality assurance through the blinded double screening process. The study is not without limitations. Because of the focus on the quality of reporting across the RE-AIM dimensions, we did not include a quality assurance tool, which would be typically seen in an efficacy-based review. It could be the case that interventions that rate low across RE-AIM in this review rate high in other reviews, or vice versa. The review could also be limited by the number of databases (five) searched and the focus on workplace interventions that measure SB as a primary outcome.

## 6. Conclusions

The results of this review indicate that there is an imbalance in the reporting of indicators across the RE-AIM framework. The improvement of reporting across all interventions, designed to reduce sedentary behavior in office workers, will be an important first step in the effective translation of interventions into real world conditions [23]. Minimal studies have been implemented at scale with substantial follow up periods, suggesting that significant barriers exist, and this fuels arguments for a more pragmatic “practice-based” approach to intervention design, in which researchers work alongside delivery agents of workplace health [20,21,121]. Regardless of the intervention design or approach, the results and subsequent recommendations of this review would provide a useful starting point for researchers in the evaluation of important, often overlooked, indicators. Improved reporting may ultimately improve the translation of research on a large scale, and have impacts on public health as intended.

## Figures and Tables

**Table 1:** Inclusion and exclusion criteria and search terms based on PICOS (population, intervention type, and comparator, outcomes of interest, and setting).

PICOS Table	Inclusion Criteria	Exclusion Criteria	Search Terms
Participants/Population	Adult office workers	Children, non-working adults, workers outside of office setting, older adults	Office staff, worksite, work *, employ *, staff, adults, white collar
Intervention	All interventions targeting SB in the workplace experimental and quasi-experimental designs, natural experiment and qualitative	Systematic reviews, meta-analysis, commentaries, conference proceedings, methodology studies, validation studies, lab-based studies	Pragmatic evaluation, process evaluation, program evaluation, feasibility, pilot, health promotion, health program, program *, trial, program theory, theory of change, logic model, health behaviour change, intervention, sitting desk, sitting workstation, cycle * workstation, treadmill desk, treadmill workstation *, active * workstation *, active * permissive workstation *, sitting workstation *, seated workstation *, height adjusted workstation *, hot desk, sit-stand desk
Comparator	All comparison or self-comparison (pre-post design, natural experiment)		
Outcome	SB measured & RE-AIM checklist elements		SB (sedentary, sedentary behave *, sedentary time, active *, sitting, sitting time, sitting behave *, screen time, screen based, chair based, deskbound, physical inactive *, inactive lifestyle, lack of activity) & RE-AIM-(Validity, external validity, internal validity, behaviour change, policy change, community change, participation, quality of life, reach, influence, effect *, success, usefulness, efficacy, adoption, acceptance, maintenance, preservation, acceptability, rate, appraise, analyses, implement, deliver *)
Setting	Office setting		

SB—sedentary behaviour; *—truncation symbol; RE-AIM: reach, efficacy/effectivness, adoption, intervention, maintenance.

**Table 2:** Characteristics of included articles.

Study Author and Year	Continent (Country)	Number of Participants	Outcome Measurement	Measurement Method	Study Type	Intervention Duration
Aittasalo et al. (2012) [29]	Europe (Finland)	*n* = 295	Primary—SB and PA Secondary—work ability and employee participation	Objective—accelerometer Subjective—workforce sitting questionnaire and additional questions on work ability	Pre- and post-longitudinal	12 months
Alkhajah et al. (2012) [30]	Australia (Australia)	*n* = 32	Primary—SB Secondary—body fat, fasting total cholesterol, HDL cholesterol, triglycerides, and glucose levels	Objective—ActivPAL, bioimpedance, and cholestech LDX analyzer	Quasi-experimental design	3 months
Arrogi et al. (2017) [31]	Europe (Belgium)	*n* = 300	Primary—SB and PA Secondary—change in health-related anthropometric measures and change in psycho-social variables	Objectively—sensewear accelerometer	Randomised control trial (RCT)	3 months
Barbieri et al. (2017) [12]	South America (Brazil)	*n* = 24	Primary—SB	Objective—monitoring sit–stand table positions	Randomised 2 group design	2 months
Ben-Ner et al. (2014) [32]	North America (USA)	*n* = 43	Primary—SB and PA Secondary—effects of work performance	Objective—Actical accelerometer Subjective—Likert scale questionnaire	RCT	12 months
Bort-Roig et al. (2014) [33]; connected to [34,35]	Europe (Spain)	*n* = 100	Primary—Update of strategies and Engagement	Subjective—semi-structured interviews and questionnaires	Mix methods	21 weeks
Brakenridge et al. (2016) [36];connected to [37]	Australia (Australia)	*n* = 50	Primary—SB Secondary—standing and moving time, reliability and validity of the LUMOback, and predictors of change.	Objective—ActivPAL	Cluster randomised trial	3 months
Brakenridge et al. (2017) [37];connected to [36]	Australia (Australia)	*n* = 50	Primary—participants perceptions of intervention	Subjective—interview and focus groups	Qualitative study	12 months
Carr et al. (2016) [38]	North America (USA)	*n* = 54	Primary—SB and PA Secondary—cardio metabolic health outcomes, musculoskeletal discomfort, and work productivity	Objective—GENEActiv accelerometer, sphygmomanometer, Subjective—WHO Health and Work Performance Questionnaire 3, Standardized Nordic Musculoskeletal Symptom Questionnaire	Two-group RCT	4 months
Carr et al. (2013) [39]	North America (USA)	*n* = 49	Primary—SB and PA Secondary—heart rate, blood pressure, height, weight, waist circumference, percent body fat, cardiorespiratory fitness, and fasting lipids	Objective—stepwatch, stethoscope, sphygmomanometer, and cholestech LDX analyzer	RCT	3 months
Carr et al. (2012) [40]	North America (USA)	*n* = 18	Primary—SB and PA	Subjective—questionnaire	Pre- and post-descriptive study	1-month
Chau, Daley, and Srinivasan et al. (2014) [41]; connected to [42]	Australia (Australia)	*n* = 42	Primary—evaluate the acceptability, feasibility, and perceptions of using sit–stand workstations	Subjective—focus groups	Qualitative	1 month
Chau, Daley, and Dunn et al. (2014) [42];connected to [41]	Australia (Australia)	*n* = 49	Primary—SB and PA	Objective—ActiGraph accelerometer Subjective—occupational sitting and physical activity questionnaire (OSPAQ)	RCT	1 month
Chau et al. (2016) [43]	Australia (Australia)	*n* = 31	Primary—SB and PA Secondary—productivity outcomes	Subjective—OSPAQ	Quasi-experimental with control	2 weeks
Cifuentes et al. (2015) [44]	North America (USA)	*n* = 5	Primary—usability, safety, comfort, and productivity using treadmill work stations in a real-world setting	Subjective—Interview and focus group	Qualitative	6 months
Coenen et al. (2017) [45]; connected to [46,47,48,49]	Australia (Australia)	*n* = 231	Primary—musculoskeletal symptoms	Subjective—27-item Nordic Musculoskeletal Questionnaire	Cross-sectional	No intervention
Coffeng et al. (2014) [50]	Europe (Netherlands)	*n* = 412	Primary—recovery experience Secondary—work-related stress, small breaks, physical activity (i.e., stair climbing, active commuting, sport activities, light/moderate/vigorous physical activity), and sedentary behaviour.	Subjective—questionnaire	RCT	12 months
Cooley et al. (2014) [14]; connected to [51]	Australia (Australia)	*n* = 47	Primary—perceptions of the outcomes associated with a workplace health intervention designed to reduce prolonged occupational sitting time	Subjective—Semi-structured interviews	Qualitative	13 weeks
Danquah IH, Kloster S, Holtermann A, Aadahl M, Tolstrup J et al. (2017) [52];connected to [53]	Europe (Denmark and Greenland)	*n* = 461	Primary—SB Secondary—musculoskeletal pain	Objective—ActiGraph Subjective—three items on pain in neck-shoulders	Cluster RCT	3 months
Danquah Danquah IH, Kloster S, Holtermann A, Aadahl M, Bauman A, Ersbøll AK, et al. (2017); [53] connected to [52]	Europe (Denmark and Greenland)	*n* = 461	Primary—SB Secondary—waist circumference and body fat percentage	Objective—ActiGraph and bioimpedance	Cluster RCT	3 months
Davis et al. (2014) [54]	North America (USA)	*n* = 37	Primary—SB, productivity discomfort	Objective—video analysis	Quasi-experimental with cross over	1 month
De Cocker et al., (2015) [55]	Europe (Belgium)	*n* = 47	Primary—SB Secondary—feasibility and acceptability	Subjective—Questionnaires	Descriptive study	2 weeks
De Cocker et al., (2016) [56]; connected to [57]	Europe (Belgium)	*n* = 213	Primary—SB Secondary—psycho-social correlates of sitting	Objective—ActivPal	RCT	3 months
De Cocker et al., (2017) [57]; connected to [56]	Europe (Belgium)	*n* = 213	Primary—SB Secondary—psycho-social correlates of sitting	Subjective—Workforce Sitting Questionnaire (WSQ)	Cluster RCT	1 month
Dewa et al. (2009) [58]	North America (Canada)	*n* = 28	Primary—SB, PA, and mental health status	Subjective—international physical activity questionnaire (IPAQ)	Quasi-experimental with control	1 month
Donath et al. (2015) [59]	Europe (Switzerland)	*n* = 38	Primary—SB Secondary—concentration, postural sway, and lower limb strength endurance	Objectively—ActiGraph	RCT	3 months
Ellegast (2012) [60]	Europe (Germany)	*n* = 25	Primary—SB and PA Secondary—health outcomes	Subjectively—Activity logs	RCT	3 months
Engelen et al. (2016) [61]	Australia (Australia)	*n* = 34	Primary—SB and PA Secondary—perceptions and productivity	Objective—accelerometer Subjective—online activity logs, mood state questionnaire, and orthopaedic medical check-up (G-46)	Natural experiment	2 months
Evans et al. (2012) [62]	Europe (U.K.)	*n* = 30	Primary—SB	Objective—ActivPAL	RCT	5 days
Fennel et al. (2016) [63]	North America (USA)	*n* = 62	Primary—SB, PA, and fitness related variables Secondary—associated psychometric factors	Subjective—IPAQ questionnaire, international personality item pool, self-efficacy and exercise habits survey, behavioural regulation in exercise questionnaire-3	RCT	4 months
Ganesan et al. (2016) [64]	Australia (Australia)	*n* = 69,219	Primary—SB and PA Secondary—weight change/BMI change and dietary change	Subjective—questionnaire	Natural experiment	100 days
Gao et al. (2016) [65]	Europe (Finland)	*n* = 45	Primary—SB Secondary—musculoskeletal discomfort and work ability	Subjective—questionnaire and Likert scale items	RCT	6 months
Gilson et al. (2009) [66]	Europe (U.K.)	*n* = 179	Primary—SB and PA	Subjective—log book	RCT	10 weeks
Gilson et al. (2016) [67]	Australia (Australia)	*n* = 57	Primary—SB	Objective—chair fitted sitting monitor	Quasi-experimental	5 months
Gorman et al. (2013) [68]	North America (Canada)	*n* = 72	Primary—SB and PA Secondary—body composition, fasting cardio-metabolic blood profile, job performance, and job satisfaction	Objective—ActivPAL	Natural experiment	4 months
Graves et al. (2015) [69]	Europe (U.K.)	*n* = 47	Primary—SB Secondary—behavioural, cardiometabolic, and musculoskeletal	Subjective—momentary assessment diary	RCT	2 months
Green et al. (2016) [70]	North America (USA)	*n* = 3	Primary—SB	Objective—ActivGraph	Pre- and post-design	NR
Hadgraft and Winkler et al. (2017) [46];connected to [45,47,48,49]	Australia (Australia)	*n* = 231	Primary—perceived behavioural control, self-efficacy, perceived organisational norms, and knowledge	Subjective—questionnaire and Adapted Likert scale single items	Qualitative study	12 months
Hadgraft and Willenberg et al. (2017) [47]; connected to [45,46,48,49]	Australia (Australia)	*n* = 136	Primary—participants’ perspectives	Subjective—semi-structured interviews	Qualitative study	12 months
Healy et al. (2017) [48];connected to [45,46,47,49]	Australia (Australia)	*n* = 231	Primary—body composition, blood pressure, glucose metabolism, lipid metabolism, and a composite overall cardiometabolic risk score	Objective	Cluster RCT	12 months
Healy et al. (2013) [71]; connected to [72]	Australia (Australia)	*n* = 43	Primary—SB Secondary—standing and stepping	Objective—ActivPAL	Non-randomised controlled trial	1 month
Healy et al. (2016) [49]; connected to [45,46,47,48]	Australia (Australia)	*n* = 231	Primary—SB Secondary—standing and stepping	Objectively—ActivPAL	RCT	12 months
Hendriksen et al. (2016) [73]	Europe (Netherlands)	*n* = 396	Primary—PA, SB, and work-related outcomes	Subjective—self-report questionnaire	Pre- and post-design—longitudinal study	5 months
Jancey et al. (2016) [74]	Australia (Australia)	*n* = 67	Primary—SB and PA	Objective—ActiGraph	Natural experimental	4 months
John et al. (2011) [75]	North America (USA)	*n* = 12	Primary—SB and PA Secondary—Health outcomes	Objective—ActivPAL	Pre- and post- design—longitudinal study	9 months
Jones et al. (2017) [76]	North America (USA)	*n* = 47	Primary—SB	Objective—Fitbit	Pre- and post-prospective cluster intervention	6 months
Judice et al. (2015) [77]	Europe (Portugal)	*n* = 10	Primary—SB Secondary—Standing and stepping	Objective—ActivPAL	RCT	1 week
Kerr et al. (2016) [78]	North America (USA)	*n* = 30	Primary—SB	Objective—ActivPAL	RCT	2 weeks
Kozey-Keadle et al. (2012) [79]	North America (USA)	*n* = 20	Primary—SB	Objective—ActivPAL	Pre- and post-design—longitudinal study	1 week
Kress et al. (2015) [80]	North America (USA)	*n* = 33	Primary—SB Secondary—personal factors and perceptions of sit–stand workstations	Subjective—questionnaire	Natural experiment	3 months
Li et al. (2017) [81]	Australia (Australia)	*n* = 33	Primary—SB Secondary—PA	Objective—ActivPAL	RCT	4 weeks
MacEwen et al. (2017) [82]	North America (Canada)	*n* = 28	Primary—SB and cardio metabolic risk factors	Objective: SB—ActivPAL Subjective: SB—non-validated questions, Cosmed Quark, Cholestech LDX system, and glycosylated haemoglobin (HbA1c) diazyme SMART analyzer	RCT	12 weeks
Mackenzie et al. (2015) [83]	Europe (U.K.)	*n* = 24	Primary—SB, and participant views	Subjective—self report sitting log, open ended question	Pre- and post-design	5 weeks
Mailey et al. (2016) [84]; connected to [85]	North America (USA)	*n* = 49	Primary—SB and cardio metabolic health	Objective SB—ActiGraph automated blood pressure cuff and Cholestech LDX	Parallel-group randomized trial	8 weeks
Mailey et al. (2017) [85];connected to [85]	North America (USA)	*n* = 49	Primary—arousal, mood, and fatigue	Subjective—activation–deactivation adjective checklist (ADACL), the positive and negative affect schedule (PANAS), and fatigue symptom inventory (FSI)	Parallel-group randomized trial	8 weeks
Mansoubi et al. (2016) [86]	Europe (U.K.)	*n* = 40	Primary—SB and PA	Objective—ActivPAL and ActiGraph accelerometer	Pre- and post- design	3 months
Neuhaus et al. (2014) [15]	Australia (Australia)	*n* = 44	Primary—SB	Objective—ActivPAL	RCT	3 months
Parry et al. (2013) [87]	Australia (Australia)	*n* = 133	Primary—SB Secondary—PA	Objective—ActiGraph accelerometer	RCT	12 weeks
Pedersen et al. (2014) [51]; connected to [14]	Australia (Australia)	*n* = 34	Primary—SB and PA	Subjective—survey built upon the OPAQ and OSPAQ	RCT	13 weeks
Priebe et al. (2015) [88]	North America (Canada)	*n* = 142	Primary—SB and PA	Subjective—Not validated SB questionnaire	Pre- and post-design	NR
Pronk et al. (2012) [89]	North America (USA)	*n* = 34	Primary—SB, health related outcomes, and work performance	Subjective—experience sampling methodology	Pre- and post-design—two groups	7 weeks
Puig-Ribera et al. (2017) [34]; connected to [33,35]	Europe (Spain)	*n* = 264	Primary—Presenteeism, productivity loss, mental well-being, and productivity	Subjective—work limitations questionnaire; Warwick–Edinburgh mental well-being scale;	Pre- and post-design—two groups	21 weeks
Puig-Ribera et al. (2015) [35]; connected to [33,34]	Europe (Spain)	*n* = 264	Primary—SB and physical risk factors for chronic disease	Subjective—self report diary log, blood pressure, weight, and waist measurement	Pre- and post- design—two groups	21 weeks
Reece et al. (2014) [90]	North America (USA)	*n* = 34	Primary—SB and PA	Objective—Sense Wear armband	RCT	17 days
Schuna et al. (2014) [91]; connected to [92]	North America (USA)	*n* = 41	Primary—SB and PA	Objective-Acti-graph	RCT	3 months
Stephens et al. (2014) [72]; connected to [71]	Australia (Australia)	*n* = 43	Primary—SB	Objective—ActivPAL	Non-randomised controlled trial	4 weeks
Straker et al. (2013) [93]	Europe (Sweden)	*n* = 131	Primary—SB	Objective—inclinometer and portable data logger	Natural experiment—cross sectional	1 day analysis
Swartz et al. (2014) [94]	North America (USA)	*n* = 78	Primary—SB and PA	Objective—ActivPAL	Randomised trial with parallel groups	2 weeks
Taylor et al. 2016 [95]	North America (USA)	*n* = 185	Primary—SB and PA	Subjective—IPAQ sitting items and self-reported seven-day checklist from the Neighbourhood Quality of Life StudyPA—pedometer and IPAQ	Cluster RCT	6 months
Tobin et al. (2016) [96]	Australia (Australia)	*n* = 52	Primary—SB Secondary—psychological distress, self-perceived physical and mental health, workability, and perceived benefits	Objective—ActivPAL Subjective—K10, SF8, and work ability index questionnaire	Pre- and post-design—two groups	5 weeks
Tudor-Lock et al. (2014) [92]; connected to [91]	North America (USA)	*n* = 41	Primary—perceptions of feasibility and acceptability	Subjective—focus groups	Qualitative	3 months
Urda et al. (2016) [97]	North America (USA)	*n* = 48	Primary—SB and perceived wellness	Objective—ActivPAL Subjective—perceived wellness survey	RCT	2 weeks
vanBerkel et al. (2014) [98]	Europe (Netherlands)	*n* = 257	Primary—SB	Subjective—non-validated SB at work questionnaire	RCT	6 months.
Venema et al. 2017 [99]	Europe (Netherlands)	*n* = 606	Primary—SB	Objective—direct observation and survey	Pre- and post-design	2 months
Verweij at al. (20d12) [100]	Europe (Netherlands)	*n* = 185	Primary—SB Secondary—PA, waist circumference, body weight, and BMI	Subjective—non-validated SB item, IPAQ Secondary outcomes—PA–(SQUASH) and BMI-calculated	RCT	6 months

NR = not reported; BMI—body mass index; HDL—high density lipoproteins; PA—physical activity.

**Table 3:** Recommendations for improved reporting across reach, effectiveness, adoption, implementation, and maintenance (RE-AIM), and examples of reporting methods used within included interventions.

RE-AIM Dimension	Recommendations for Improved Reporting across the RE-AIM Framework for Interventions Targeting Sedentary Behaviour in Office Workers
Reach	Seek or collect basic demographic or health information of all workplace setting employees, which will help to compare participants vs. non-participants. Example method found in De Cocker et al. (2016) and De Cocker et al. (2017) [56,57]. Report the number of participants exposed to recruitment activities and illustrate the calculation of participation rate of the study. Employ questionnaire or qualitative methods to understand barriers to reach of study. Example method found in Bort-Riog et al. (2014) [33].
Effectiveness	If intention to treat methods are used, report specific method and rationale for appropriateness. Example method found in Arrogi et al. (2017) [31]. Seek to use biological outcome measures (e.g., body composition, cardiovascular fitness, glucose metabolism and overall cardiomtabolic risk score). Example methods found in Healy et al. (2017) [48]. Use questionnaire and/or qualitative methods to understand impact on quality of life and unintended or unexpected outcomes. Example methods found in Pronk et al. (2012) [89] and Hardgraft et al. (2017) [47]. Additional questionnaires utilised for unexpected outcomes including: musculoskeletal (27-item Nordic musculoskeletal questionnaire), presenteeism (work limitations questionnaire (WLQ), percentage of work productivity loss (WLQ index score) and mental well-being (Warwick–Edinburgh mental well-being scale (WEMWBS)). Productivity—the work limitations questionnaire (WLQ) assessed profile of mood states (POMS) questionnaire.
Adoption	Record and report on the specific recruitment processes, including: inclusion and exclusion criteria for businesses, the number of companies or sites approached, the number who declined available demographic information to report on representativeness of company demographics compared to local area statistics (e.g., state or province or council demographic statistics.). Example method found in Puig-Ribera et al. (2015) [35]. Collect quantitative information from implementation team regarding level of training and expertise and fidelity to implementation strategies. Example method found in Brakenridge et al. 2017 [37] Aittasalo et al. (2012) [29]. Report a measure of cost to implement per setting.
Implementation	Collect qualitative or questionnaire data from the implementation team regarding the fidelity to implementation strategies and facilitators and barriers to implementation. Example method found in Bort-Riog et al. (2014) [33]. Collect qualitative or questionnaire data regarding facilitators and barriers to uptake of behaviour change strategies. Example method found in Bort-Riog et al. (2014) [33]. Report on cost (monetary or time commitment) of implementation of individual intervention strategies.
Maintenance	Record and report plans for follow-up and any modifications to program. Utilise accessible questionnaire’s from which to collect data at more long-term follow-up time points. Example methods such as self-report logs or sitting items from existing questionnaires found in Coffeng et al. (2014) [50], Gao et al. (2016) [65], and van Berkel et al. (2014) [98].

## Supplementary Materials

Supplementary materials are available online at http://www.mdpi.com/1660-4601/15/12/2876/s1. Table S1: reporting of indicators of reach and effectiveness, across all of the included interventions, Table S2: reporting of indicators of adoption, implementation, and maintenance, across all of the included interventions.
