# Supplementary material for: An Integrative, Systematic Review Exploring the Research, Effectiveness, Adoption, Implementation, and Maintenance of Interventions to Reduce Sedentary Behaviour in Office Workers
Source: Int J Environ Res Public Health. 2018 Dec 15;15(12):2876. doi: 10.3390/ijerph15122876 (PMC6313589; doi:10.3390/ijerph15122876)
Supplement: Supplementary file 1 [file ijerph-15-02876-s001.zip › Supplementary table 1. Reporting of indicators of reach and effectiveness across all included interventions.docx]

**Supplementary table 1. Reporting of indicators of reach and effectiveness across all included interventions**

|  | REACH | | | | | | | EFFICACY/EFFECTIVENESS | | | | | |
| --- | --- | --- | --- | --- | --- | --- | --- | --- | --- | --- | --- | --- | --- |
| Study Author & Year | Method to identify the target population | Inclusion criteria | Exclusion criteria | Sample size | Participation rate | Use of qualitative methods to understand the reach or recruitment | Characteristics of participants | Measure/results (at shortest assessment) | Imputation procedures (created data) | Quality of life measurements | Effects at longest (extra follow up) | Use of qualitative methods or data to understand outcomes | Per cent attrition rate (drop out rate) |
| Aittasalo et al. (2012) [29] | ✓ | ✓ | ✓ | ✓ | ✓ |  |  | ✓ |  |  | ✓ |  | ✓ |
| Alkhajah et al. (2012) [30] | ✓ | ✓ | ✓ | ✓ |  |  |  | ✓ |  |  | ✓ |  |  |
| Arrogi et al. (2017) [31] | ✓ | ✓ |  | ✓ |  |  |  | ✓ | ✓ |  | ✓ |  | ✓ |
| Barbieri et al. (2017) [12] |  | ✓ |  | ✓ |  |  |  | ✓ |  |  |  |  |  |
| Ben-Ner et al. (2014) [32] | ✓ |  | ✓ | ✓ | ✓ |  |  | ✓ |  |  | ✓ |  | ✓ |
| Bort-Roig et al. (2014) [33]  connected to Puig-Ribera et al. (2015) [88], Puig-Ribera et al. (2017)[87] | ✓ | ✓ | ✓ | ✓ | ✓ | ✓ |  | ✓ |  | ✓ | ✓ |  | ✓ |
| Brakenridge et al. (2016) [34] connected to  Brakenridge et al. (2017) [35] | ✓ | ✓ | ✓ | ✓ | ✓ | ✓ |  | ✓ | ✓ | ✓ | ✓ | ✓ | ✓ |
| Carr et al. (2016) [36] | ✓ |  | ✓ | ✓ | ✓ |  |  | ✓ |  |  | ✓ |  | ✓ |
| Carr et al. (2013) [37] | ✓ | ✓ | ✓ | ✓ | ✓ |  |  | ✓ |  |  | ✓ |  | ✓ |
| Carr et al. (2012) [38] | ✓ | ✓ | ✓ | ✓ |  |  |  | ✓ |  | ✓ | ✓ |  |  |
| Chau, Daley & Srinivasan et al. (2014) [39] Connected to Chau, Daley & Dunn et al. (2014) [40] | ✓ | ✓ |  | ✓ | ✓ |  |  | ✓ | ✓ |  | ✓ | ✓ | ✓ |
| Chau et al. (2016) [41] | ✓ |  |  | ✓ |  |  |  | ✓ |  |  | ✓ |  | ✓ |
| Cifuentes et al. (2015) [42] | ✓ |  |  | ✓ | ✓ | ✓ |  | ✓ |  | ✓ |  | ✓ |  |
| Coenen et al. (2017) [43]  Connected to Hadgraft NT, Willenberg L, LaMontagne AD, Malkoski K, Dunstan DW, Healy GN, et al. (2017) [64]; Hadgraft NT, Winkler EA, Healy GN, Lynch BM, Neuhaus M, Eakin EG, et al. (2017) [65]; Healy GN, Eakin EG, LaMontagne AD, Owen N, Winkler EA, Wiesner G, et al. (2017) [66]; Healy GN, Eakin EG, Owen N, Lamontagne AD, Moodie M, Winkler E, et al. (2016) [68]. | ✓ | ✓ | ✓ | ✓ |  |  | ✓ | ✓ | ✓ | ✓ | ✓ | ✓ | ✓ |
| Coffeng et al. (2014) [44] | ✓ | ✓ | ✓ | ✓ | ✓ |  |  | ✓ |  |  | ✓ |  | ✓ |
| Cooley et al. (2014) [14]  connected to Pedersen et al. (2014) [84] | ✓ | ✓ |  | ✓ | ✓ |  |  | ✓ |  | ✓ |  | ✓ |  |
| Danquah IH, Kloster S, Holtermann A, Aadahl M, Tolstrup JSJSjow et al. (2017) [45]  connected to Danquah Danquah IH, Kloster S, Holtermann A, Aadahl M, Bauman A, Ersbøll AK, et al. (2017) [46] | ✓ |  | ✓ | ✓ |  |  |  | ✓ | ✓ | ✓ | ✓ |  | ✓ |
| Davis et al. (2014) [47] | ✓ | ✓ | ✓ | ✓ | ✓ |  |  | ✓ |  | ✓ | ✓ |  | ✓ |
| De Cocker et al., (2015)[48] | ✓ | ✓ | ✓ | ✓ | ✓ |  | ✓ | ✓ |  |  | ✓ |  | ✓ |
| De Cocker et al., (2016) [49]  connected to De Cocker et al., (2017) [50] | ✓ | ✓ | ✓ | ✓ | ✓ |  |  | ✓ |  |  | ✓ |  | ✓ |
| Dewa et al. (2009) [51] | ✓ |  |  | ✓ |  |  |  | ✓ |  |  | ✓ |  |  |
| Donath et al. (2015) [52] | ✓ | ✓ | ✓ | ✓ | ✓ |  |  | ✓ |  |  | ✓ |  | ✓ |
| Ellegast. (2012) [53] | ✓ |  |  | ✓ |  |  |  | ✓ |  |  | ✓ |  |  |
| Engelen et al. (2016) [54] | ✓ |  |  | ✓ |  |  |  | ✓ |  |  | ✓ |  | ✓ |
| Evans et al. (2012) [55] | ✓ | ✓ | ✓ | ✓ |  |  |  | ✓ |  |  | ✓ |  | ✓ |
| Fennel et al. (2016) [56] | ✓ | ✓ | ✓ | ✓ | ✓ |  |  | ✓ |  |  | ✓ |  |  |
| Ganesan et al. (2016) [57] | ✓ | ✓ | ✓ | ✓ | ✓ |  |  | ✓ |  |  | ✓ |  | ✓ |
| Gao et al. (2016) [58] | ✓ | ✓ | ✓ | ✓ |  |  |  | ✓ |  |  | ✓ |  | ✓ |
| Gilson et al. (2009) [59] | ✓ |  |  | ✓ | ✓ |  |  | ✓ | ✓ |  | ✓ |  |  |
| Gilson et al. (2016) [60] | ✓ | ✓ | ✓ | ✓ | ✓ |  |  | ✓ |  |  | ✓ |  |  |
| Gorman et al. (2013) [61] | ✓ | ✓ | ✓ | ✓ | ✓ |  |  | ✓ |  |  | ✓ |  | ✓ |
| Graves et al. (2015) [62] | ✓ | ✓ | ✓ | ✓ | ✓ |  |  | ✓ | ✓ |  | ✓ |  | ✓ |
| Green et al. (2016) [63] | ✓ | ✓ |  | ✓ | ✓ |  |  | ✓ |  |  |  |  | ✓ |
| Healy et al. (2013) [67] connected to Stephens et al. (2014) [91] | ✓ | ✓ | ✓ | ✓ |  |  |  | ✓ |  |  | ✓ |  |  |
| Hendriksen et al. (2016) [69] | ✓ | ✓ | ✓ | ✓ | ✓ |  |  | ✓ |  |  | ✓ |  | ✓ |
| Jancey et al. (2016) [70] | ✓ | ✓ | ✓ | ✓ | ✓ |  |  | ✓ |  |  | ✓ |  | ✓ |
| John et al. (2011) [71] | ✓ | ✓ | ✓ | ✓ |  |  |  | ✓ |  |  | ✓ |  | ✓ |
| Jones et al. (2017) [72] | ✓ | ✓ | ✓ | ✓ | ✓ |  | ✓ | ✓ |  |  |  |  | ✓ |
| Judice et al. (2015) [73] | ✓ | ✓ |  | ✓ | ✓ |  |  | ✓ |  |  | ✓ |  | ✓ |
| Kerr et al. (2016) [74] | ✓ | ✓ | ✓ | ✓ |  |  |  | ✓ | ✓ |  | ✓ |  |  |
| Kozey-Keadle et al. (2012) [75] | ✓ | ✓ | ✓ | ✓ |  |  |  | ✓ |  |  | ✓ |  |  |
| Kress et al. (2015) [76] | ✓ | ✓ |  | ✓ | ✓ |  |  | ✓ |  |  |  |  | ✓ |
| Li et al. (2017) [77] | ✓ | ✓ |  | ✓ | ✓ |  |  | ✓ |  |  |  |  | ✓ |
| MacEwen et al. (2017) [78] | ✓ | ✓ | ✓ | ✓ |  |  |  | ✓ |  | ✓ |  |  | ✓ |
| Mackenzie et al. (2015) [79] | ✓ |  |  | ✓ | ✓ |  | ✓ | ✓ |  |  |  | ✓ | ✓ |
| Mailey et al. (2016) [80] connected to Mailey et al. (2017) [81] | ✓ | ✓ | ✓ | ✓ | ✓ |  |  | ✓ |  | ✓ |  |  | ✓ |
| Mansoubi et al. (2016) [82] | ✓ | ✓ | ✓ | ✓ |  |  |  | ✓ |  |  | ✓ |  | ✓ |
| Neuhaus et al. (2014) [15] | ✓ | ✓ |  | ✓ | ✓ |  |  | ✓ |  | ✓ |  |  | ✓ |
| Parry et al. (2013) [83] | ✓ | ✓ | ✓ | ✓ |  |  | ✓ | ✓ |  |  |  |  | ✓ |
| Priebe et al. (2015) [85] |  | ✓ |  | ✓ |  |  |  | ✓ |  |  |  |  | ✓ |
| Pronk et al. (2012) [86] |  | ✓ |  | ✓ | ✓ |  |  | ✓ |  | ✓ |  |  |  |
| Reece et al. (2014) [89] | ✓ | ✓ |  | ✓ |  |  |  | ✓ |  |  |  |  | ✓ |
| Schuna et al. (2014) [90] connected to Tudor-Lock et al. (2014) [96] | ✓ | ✓ | ✓ | ✓ | ✓ | ✓ |  | ✓ |  | ✓ |  | ✓ | ✓ |
| Straker et al. (2013)[92] | ✓ | ✓ |  | ✓ | ✓ |  |  | ✓ |  |  |  |  | ✓ |
| Swartz et al. (2014) [93] | ✓ | ✓ | ✓ | ✓ | ✓ |  |  | ✓ |  |  |  |  | ✓ |
| Taylor et al. (2016) [94] | ✓ | ✓ |  | ✓ | ✓ |  | ✓ | ✓ |  |  |  |  | ✓ |
| Tobin et al. (2016) [95] | ✓ | ✓ |  | ✓ |  |  |  | ✓ |  | ✓ |  |  | ✓ |
| Urda et al. (2016) [97] | ✓ | ✓ | ✓ | ✓ | ✓ |  |  | ✓ |  | ✓ |  |  | ✓ |
| vanBerkel et al. (2014) [98] | ✓ | ✓ |  | ✓ | ✓ |  |  | ✓ | ✓ |  | ✓ |  | ✓ |
| Venema et al. (2017) [99] |  |  |  | ✓ |  |  |  | ✓ |  |  | ✓ |  | ✓ |
| Verweij at al. (2012) [100] | ✓ | ✓ | ✓ | ✓ |  |  |  | ✓ |  |  |  |  | ✓ |
